# Establishment of a developmental toxicity assay based on human iPSC reporter to detect FGF signal disruption

**DOI:** 10.1016/j.isci.2022.103770

**Published:** 2022-01-15

**Authors:** Seiya Kanno, Yusuke Okubo, Tatsuto Kageyama, Lei Yan, Satoshi Kitajima, Junji Fukuda

**Affiliations:** 1 Faculty of Engineering, Yokohama National University, 79-5 Tokiwadai, Hodogaya Ward, Yokohama, Kanagawa 240-8501, Japan; 2 Division of Cellular & Molecular Toxicology, Center for Biological Safety & Research, National Institute of Health Sciences, 3-25-26 Tono-machi, Kawasaki-ku, Kawasaki, Kanagawa 210-9501, Japan; 3 TechnoPro, Inc., 6-10-1 Roppongi, Minato City, Tokyo 106-6135, Japan; 4 Kanagawa Institute of Industrial Science and Technology (KISTEC), 3-2-1 Sakado, Takatsu Ward, Kawasaki, Kanagawa 213-0012, Japan

**Keywords:** Pharmacological parameters, Toxicology evaluation, Toxicity assessment, Embryology

## Abstract

The number of man-made chemicals has increased exponentially recently, and exposure to some of them can induce fetal malformations. Because complex and precisely programmed signaling pathways play important roles in developmental processes, their disruption by external chemicals often triggers developmental toxicity. However, highly accurate and high-throughput screening assays for potential developmental toxicants are currently lacking. In this study, we propose a reporter assay that utilizes human-induced pluripotent stem cells (iPSCs) to detect changes in fibroblast growth factor signaling, which is essential for limb morphogenesis. The dynamics of this signaling after exposure to a chemical were integrated to estimate the degree of signaling disruption, which afforded a good prediction of the capacity of chemicals listed in the ECVAM International Validation Study that induce limb malformations. This study presents an initial report of a human iPSC-based signaling disruption assay, which could be useful for the screening of potential developmental toxicants.

## Highlights

• Human iPSC-based FGF signal disruption reporter system was established
• FGF signal disruption was a good indicator of limb malformation-related toxicants
• Integration of dynamic FGF signal disruption results improved assay performance

## Graphical abstract

**Figure:**
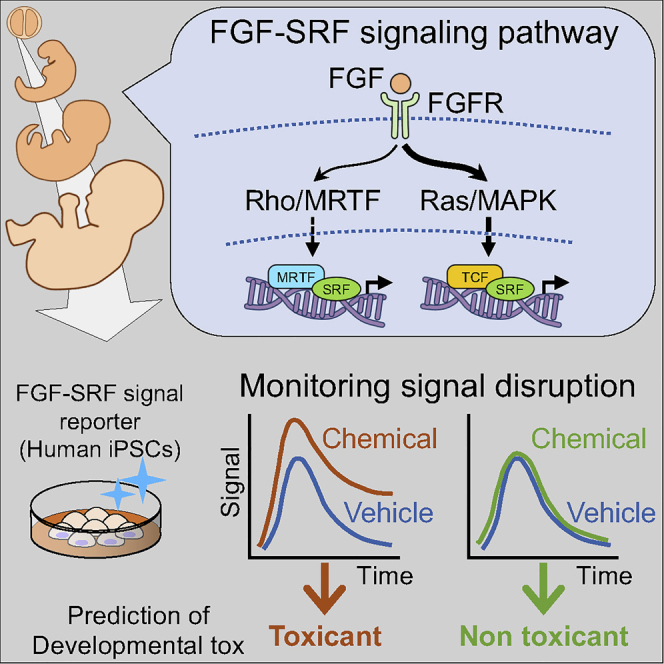

## Introduction

Approximately 7.9 million babies, which comprise 6% of the world’s birth population, are born annually with some form of congenital malformations (Christianson et al., 2006) that often require surgical treatment. Malformations are caused not only by genetic factors but also by the exposure to environmental factors, such as teratogenic chemicals, during fetal organogenesis. The tragedy with thalidomide in the 1950s and early 1960s highly influenced the official regulation for the use of potential developmental toxicants to which pregnant women may be exposed. Developmental toxicity studies have been conducted on pregnant animals by exposing them to chemicals such as industrial compounds, food additives, and agricultural pesticides. This approach detects the deleterious impact of such chemicals on the fetal development of multiple organs. However, animal testing requires a large number of animals, involves costly procedures, and is time-consuming. Furthermore, there is a worldwide effort to implement the 3R (replacement, reduction, and refinement) principles for animal protection (van der Laan et al., 2012). Therefore, inexpensive, high-throughput, and highly predictive *in vitro* developmental toxicity assays are necessary.

There have been several reports on *in vitro* developmental toxicity assays based on differentiation markers of various tissues, including cardiomyocytes (Suzuki et al., 2011) and neurons (Kobayashi et al., 2017). This approach can be used for screening developmental toxicants *in vitro*, but in principle, detection is limited to the influence on specific tissues. It requires various differentiation culture systems and a series of molecular markers for testing the developmental toxicity toward different tissues and organs. During embryogenesis, several major signaling cascades, such as fibroblast growth factor (FGF), Hedgehog, Wnt, transforming growth factor ß (TGFβ), and Notch pathways, are repeatedly utilized in various tissues at different stages to induce programmed cellular responses for precise morphogenesis (Sanz-Ezquerro et al., 2017). Therefore, disruption of such signaling pathways could be a better and more comprehensive indicator for testing systemic developmental toxicity. For example, cadmium chloride interferes with the phosphorylation of extracellular signal-regulated kinase **(**Erk) 1/2, an effector of the FGF signaling pathway, and induces limb malformation (Elsaid et al., 2007). Thalidomide binds to cereblon and alters its substrate specificity, which promotes the ubiquitination of various proteins and eventually decreases the expression of FGF8 and other proteins important for limb formation (Asatsuma-Okumura et al., 2019). However, highly accurate and high-throughput screening assays using signal disruption by developmental toxicants are currently lacking.

Receptor tyrosine kinases (RTKs) belong to a family of cell surface receptors that bind various growth factors, including FGF, epidermal growth factor (EGF), and vascular endothelial growth factor. They are involved in almost all stages of embryonic development, from early tissue patterning to organogenesis (Lemmon and Schlessinger, 2010). For example, the FGF interacts with FGFRs, members of the RTK family, activating a signaling pathway that plays an important role in the formation of the limbs, palate, and vertebrae during morphogenesis (Morgani et al., 2018). A major transcription factor downstream of the RTK signaling pathway is the serum response factor (SRF) (Vasudevan and Soriano, 2014). Tissue-specific SRF knockout experiments have clearly demonstrated an important role of SRF in developmental processes. Mutating *Srf* specifically in vascular endothelial cells of mouse embryos caused vascular defects mainly in the limb buds, head, and tail, eventually leading to embryonic lethality at E14.5 (Franco et al., 2008). Similarly, mutating *Srf* specifically in mouse neural crest cells caused craniofacial clefts owing to impaired proliferation and migration of cranial neural crest cells and their derivatives (Vasudevan and Soriano, 2014). These studies suggest that the disruption of the RTK/SRF signaling pathway can be a good indicator of the developmental toxicity of chemicals.

The objective of this study was to utilize recent technical advances in genome-editing and human-induced pluripotent stem cell (iPSC) research and develop a live-cell luciferase assay system for the monitoring time course of changes in the activity of the RTK/SRF signaling pathway via FGF-activated FGFRs. We generated and optimized this initial model system and validated its performance using 18 test chemicals, including 7 limb malformation-inducing chemicals, selected from a report about the ECVAM International Validation Study (Genschow et al., 2002). Our data provide evidence that our human iPSC-based signaling disruption reporter assay system is a promising tool for screening developmental toxicants.

## Results

### Gene editing of human human-induced pluripotent stem cells to generate receptor tyrosine kinases/serum response factor signal reporter cells

**Figure 1:**
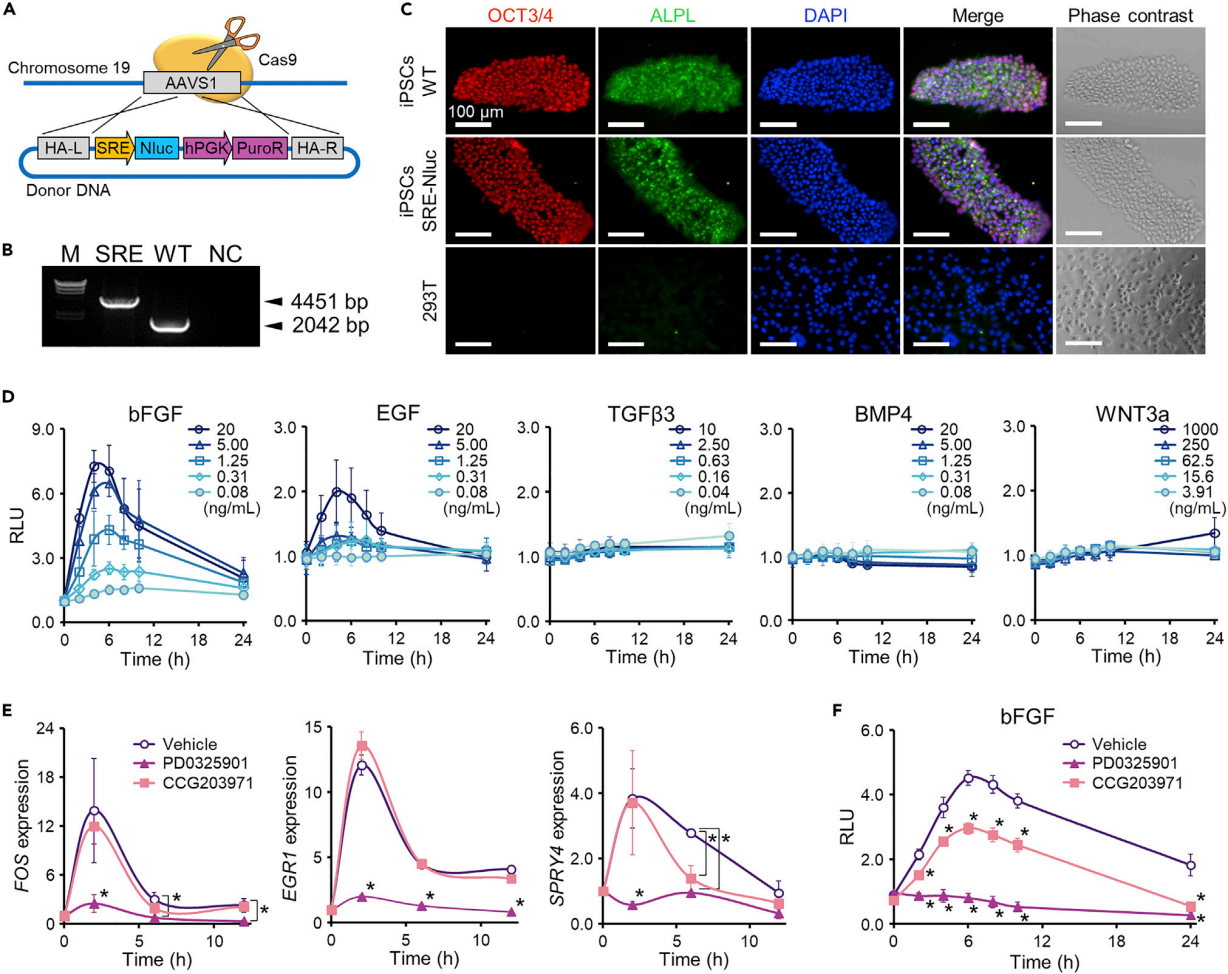
Generation of RTK/SRF signal reporter cells from human iPSCs and their characterization (A) Schematic of targeted transgene insertion into the *AAVS1* locus in the human genome. The donor vector contains *AAVS1* homology arms about 800 bp long at both ends of the donor plasmid for efficient knock-in. The construct plasmid contains the nano-luciferase (*Nluc*) reporter gene under the control of the SRF response element (SRE) and the puromycin resistance gene (*PuroR*) under the control of the human phosphoglycerate kinase (hPGK) promoter. (B) Genome PCR analysis of RTK/SRF signal reporter cells confirming donor DNA knock-in. (C) Immunofluorescence detection of pluripotent stem cell-specific markers OCT4 (red) and ALPL (green) in wild-type human iPSCs and RTK/SRF signal reporter cells. Nuclei were stained with 4′,6-diamidino-2-phenylindole (DAPI, blue). (D) Dynamics of the ligand-responsive live-cell luciferase activity in RTK/SRF signal reporter cells. Cells were treated with several ligands (bFGF, EGF, TGFβ3, BMP4, and WNT3A) at different concentrations. Luminescence intensity data are presented as the mean ± standard deviation (SD) relative to those in vehicle control at each time point (n = 3). (E) Alterations in the transcriptional activity of FGF-regulated genes by specific signal transduction inhibitors. Human unmodified iPSCs were treated with the Ras/MAPK inhibitor 1 μM PD0325901 or the Rho/MRTF inhibitor 10 μM CCG203971 for 1 h (−1 h), followed by the treatment with bFGF for 12 h. Data are presented as the mean ± S.D. of the relative expression level normalized by the *GAPDH* signal at each time point and then to the level prior to bFGF addition (defined as 0 h, n = 3). Asterisks (∗) indicate data points significantly different from vehicle control points (p < 0.05, two-way ANOVA followed by the Bonferroni multiple comparison test). (F) Alterations in the live-cell luciferase activity in FGF/SRF signal reporter cells caused by PD0325901 and CCG203971 applied 1 h before the treatment with bFGF. The luminescence intensity was normalized to that of the vehicle control group (PBS + 0.1% BSA + vehicle for inhibitors). Data are presented as the mean ± S.D. (n = 3). Statistical analysis was performed as described in (E)

To monitor changes in the activity of the RTK/SRF signaling pathway, reporter cells were prepared by the genome-editing insertion of the NanoLuc luciferase gene (*Nluc*) under the control of the serum response element (SRE) (Figure 1A). Nluc is an ATP-independent small enzyme engineered from the luciferase of deep-sea shrimp (Hall et al., 2012). Upon binding its synthetic substrate, Nluc generates luminescent signals that are >100 times brighter than signals of luciferases from other species. The sensitivity coupled with the stability of the enzyme made the Nluc system an ideal candidate for live-cell luciferase assays. SRE is a DNA-binding site for SRF downstream of RTK signaling. The plasmid vectors were constructed by incorporating SRE, *Nluc*, and puromycin resistance genes between the left and right homology arms. The donor genes were then knocked into the *AAVS1* region in the human iPSC genome using CRISPR/Cas9 genome-editing technology to avoid interference with host gene expression (DeKelver et al., 2010). After puromycin selection (Figures S1A and S1B), homozygous insertion of the genes was confirmed by the presence of the PCR (PCR) band approximately 2.5 kb longer than that of the wild-type signal (Figure 1B). Additionally, Sanger sequencing of PCR products showed that donor genes were correctly knocked in without any indel mutations (Figure S1C). Immunostaining for OCT3/4 and ALPL revealed that the reporter cells remained undifferentiated after the preparation process (Figure 1C).

Next, responses of the reporter cells to several ligands (bFGF, EGF, TGFβ3, BMP4, and WNT3A) were examined (Figure 1D). The time course of changes in the luminescence intensity was determined over 24 h after the addition of each ligand. The intensity significantly increased and reached a peak at approximately 4–6 h after the addition of bFGF or EGF. The increase in the intensity was highly dependent on the ligand concentration. There was little change in the intensity after the addition of TGFβ3, BMP4, or WNT3A. The differences in the intensity change induced by the ligands may be attributed to the differences in expression levels of relevant receptors, intracellular signal cascade molecules, and transcription factors (Gualdrini et al., 2016; Mack, 2011; Olson and Nordheim, 2010), but the exact molecular mechanisms underlying the diversity of responses were outside the scope of our study. Most importantly, the reporter cells responded to bFGF, which encouraged us to use inhibitors of the FGF signaling pathway in the next series of experiments.

SRF regulates the expression of various genes by forming a complex with the ternary complex factor (TCF) or myocardin-related transcription factor (MRTF). TCF and MRTF are located downstream of the Ras/MAPK pathway and Rho/MRTF pathway, respectively (Gualdrini et al., 2016; Vasudevan and Soriano, 2014). The reporter cells were pre-treated with the inhibitors of the Ras/MAPK pathway (PD0325901) and Rho/MRTF pathway (CCG203971) 1 h before the treatment with bFGF, and the expression of genes encoding downstream components of the FGF signaling pathway (*FOS*, *EGR1*, and *SPRY4*) was analyzed by RT-qPCR (Figure 1E). PD0325901 significantly suppressed the expression of these genes, whereas no suppression was observed following pre-treatment with CCG203971, except for the suppression of the *SPRY4* mRNA level at 6 h. The same inhibition experiments using reporter cells showed that the luminescence intensity was completely and moderately suppressed by PD0325901 and CCG203971, respectively. The observed changes in gene expression and luminescence suggest that the Ras/MAPK pathway is dominant in the FGF-activated RTK/SRF signaling cascade. This is consistent with a previous report that described the dependence of the dominant pathway on RTK ligands and the dominance of the Ras/MAPK and Rho/MRTF pathways in the FGF- and PDGF-activated RTK/SRF signaling cascades, respectively (Vasudevan and Soriano, 2014). Our results indicate that the prepared reporter cells can be used to monitor the activity of the FGFR-mediated RTK/SRF signaling pathway.

### Detection of developmental toxicants based on the disruption of the fibroblast growth factor signaling pathway

The chemicals tested in this study are listed in Table 1. Eighteen commercially available chemicals were selected from the 20 chemicals used in the ECVAM validation study of embryotoxicity tests *in vitro* (Genschow et al., 2002). According to the EVCAM study, 12 and 6 of our selected chemicals were embryotoxic and non-embryotoxic, respectively. Given that FGF signaling is closely associated with limb morphogenesis (Ornitz and Itoh, 2015), we categorized 7 out of the 12 toxic chemicals by their involvement in limb malformation, based on the appearance frequency of their chemical names in publications with the keywords “limb malformation” and “developmental toxicity” in the PubMed database (Table S1). Limb malformation by the seven chemicals was further confirmed in published animal experiments (Table S2) (Campbell et al., 2004, Haryono et al., 2011, Hyoun et al., 2012, Joschko et al., 1993, Okuda et al., 1997, Paradis and Hales, 2013, Rodríguez-Pinilla et al., 2000, Schlisser and Hales, 2013, Tanaka et al., 1973).

**Table 1:** Tested chemicals from the ECVAM International Validation Study on embryotoxicity tests *in vitro*, their vehicles, and maximum concentrations used in this study

Developmental toxicity	Test chemicals	Abbreviation	CAS No.	Vehicle	Max Conc. (μg/mL)
Positive	all-*trans*-Retinoic acid	ATRA	302-79-4	DMSO	0.010
	Hydroxyurea	HU	127-07-1	PBS	149
	Methoxyacetic acid	MAA	625-45-6	PBS	683
	Methylmercury chloride	MeHg	115-09-3	DMSO	1.20
	Methotrexate hydrate	MTX	133073-73-1	DMSO	5.00
	Sodium salicylate	SA	54-21-7	PBS	666
	Valproic acid	VPA	99-66-1	DMSO	100
	6-Aminonicotinamide	6-AN	329-89-5	DMSO	1.00
	Boric acid	BA	10043-35-3	PBS	250
	5-Bromo-2′-deoxyuridine	BrdU	59-14-3	DMSO	50.0
	5,5-Dimethyl-2,4-oxazolidinedione	DMO	695-53-4	PBS	840
	Lithium chloride	LiCl	7447-41-8	PBS	250
Negative	Acrylamide	AcA	79-06-1	PBS	454
	D-Camphor	CAM	464-49-3	DMSO	50.0
	Diphenhydramine hydrochloride	DHM	147-24-0	PBS	262
	Dimethyl phthalate	DMP	131-11-3	DMSO	100
	Penicillin G sodium salt	PenG	69-57-8	PBS	1,000
	Sodium saccharin	SAC	82385-42-0	PBS	1,000

The underlined values indicate IC_50_ estimated by the cell survival assay. See also Figure S2. DMSO, dimethyl sulfoxide; Max conc, maximum concentration, PBS, phosphate-buffered saline.

**Figure 2:**
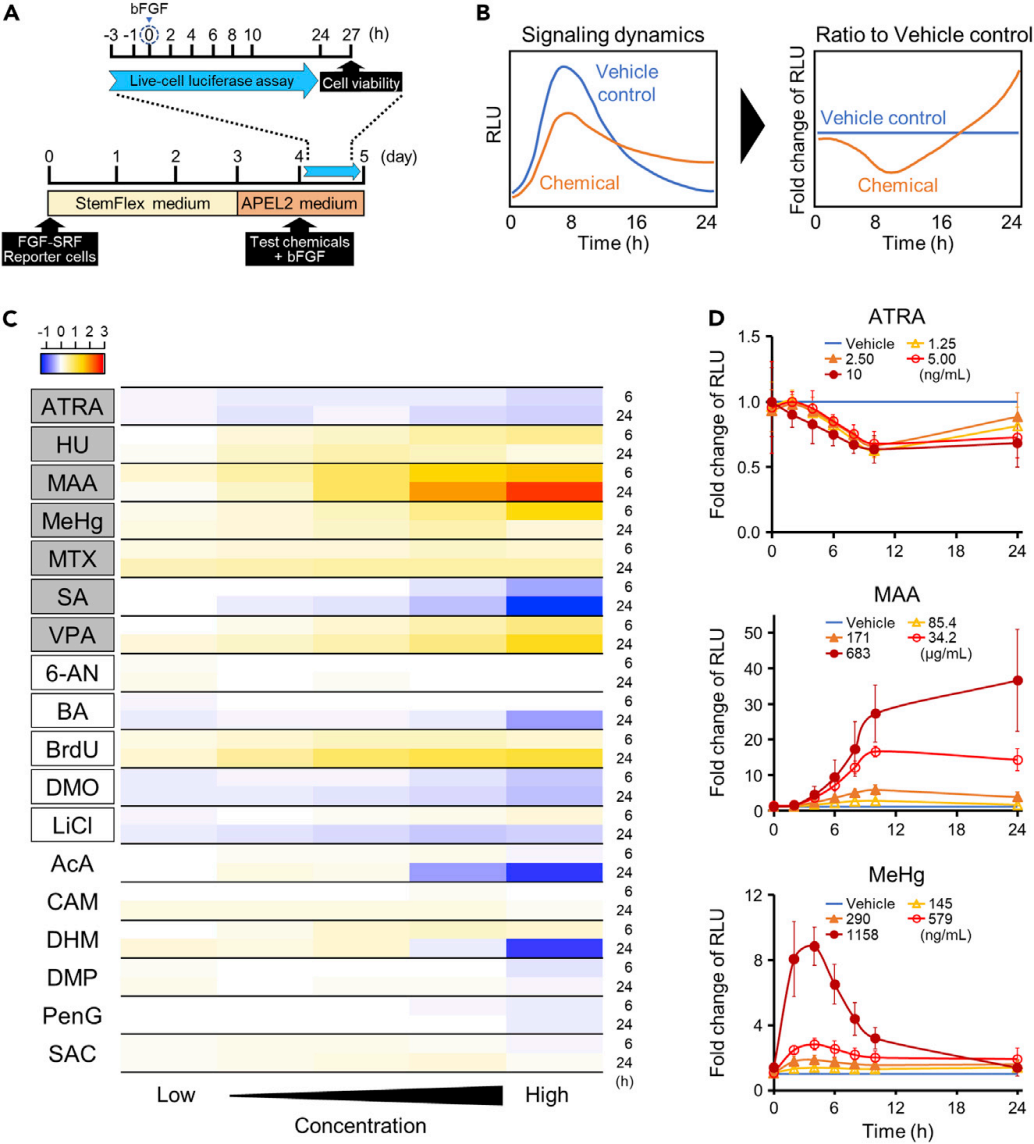
Time course of changes in the extent of signaling disruption by the chemicals tested (A) Experimental procedures. FGF/SRF signal reporter cells were exposed to chemicals at different concentrations or to the vehicle 1 h before (−1 h) the treatment with bFGF (0 h). The live-cell luciferase assay was performed over 24 h (0–24 h), and cell viability was measured 3 h later (27 h). (B) Schematics of the normalization of the luminescence intensity time course. (C) Heatmap of the luminescence intensity at 6 and 24 h after the treatment with bFGF. The intensity was normalized and log-transformed. The names of the developmental toxicants are written within a box. The toxicants causing limb malformation are labeled with gray color. (D) Representative changes in the luminescence intensity following the exposure to three selected chemicals. Data are presented as the mean ± S.D. (n = 3)

To determine the range of exposure concentrations of the 18 chemicals, we examined the dependence of reporter cell viability on the concentration of each chemical (Figure S2). The maximum exposure concentration of each chemical was set to either the IC_50_ value, using the viability curve (Genschow et al., 2002; Le Coz et al., 2015), or the maximum soluble concentration in the culture medium at which the viability remained above 50% (Table 1). To quantify the disruption of the FGF signaling pathway, the reporter cells were cultured in the maintenance medium for 3 days and in the bFGF-depleted medium for 1 day. After a 1 h exposure to chemicals (−1 h) followed by the stimulation with bFGF (0 h), the luminescence intensity was monitored over a period of 24 h (Figures 2A and S3–S5). After monitoring, cell viability was measured after 3 h to confirm that it was above 50% (Figures S3–S5). The luminescence intensity was normalized to that of the vehicle control group at the corresponding time points (Figure 2B). The heatmap of log-transformed fold changes in the intensity at 6 and 24 h after FGF stimulation clearly indicates that the signal activity changed over time regardless of whether the cells were exposed to developmental toxicants or non-toxicants (Figure 2C). LiCl, acrylamide (AcA), and diphenhydramine hydrochloride (DHM) showed opposite (positive/negative) effects on signal disruption at 6 and 24 h. Mechanisms responsible for this phenomenon were not elucidated in this study, but it might be attributed to the feedback loop and amplification of bypass pathways in complex intracellular signaling systems. Thus, the estimation of the toxicity based on the measurements at a single time point may lead to the misidentification of developmental toxicants. Representative time courses of changes in the intensity of luminescence following the exposure to three chemicals known to cause limb malformation, all-*trans*-retinoic acid (ATRA), methoxyacetic acid (MAA), and methylmercuric chloride (MeHg), are shown in Figure 2D. ATRA and MAA negatively and positively affected the FGF signaling pathway, respectively. Furthermore, the dynamics of the disruption were quite different among the chemicals. The exposure to ATRA and MAA tended to increase the extent of the disruption for up to 10 h and maintain the dysregulated level thereafter, whereas the exposure to MeHg caused maximal disruption early, at ∼4 h, and then, the extent of the disruption decreased. These results indicate that focusing on single endpoint measurements in the signaling reporter assay could be misleading, and we next examined whether monitoring dynamic changes in signaling activity would provide a more precise detection of toxic properties.

### Prognostic value of the area between the curves (area between the curves) parameter in the live-cell luciferase assay

**Figure 3:**
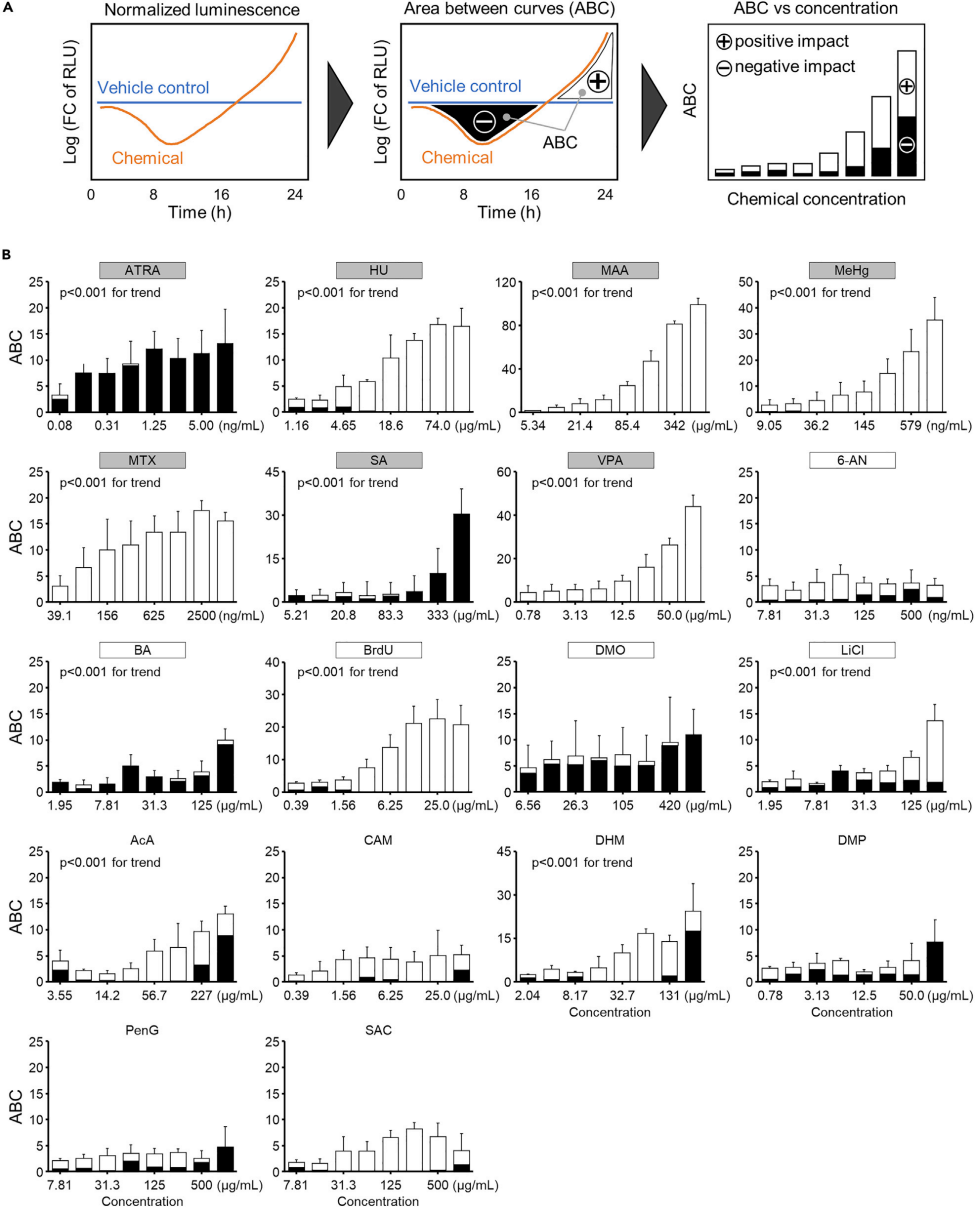
Area between curves (ABC) as a measure of signaling disruption magnitude (A) Integration of ABC of the iPSCs exposed to the vehicle control and the chemical. Positive and negative ABC areas were accumulated to express the extent of disruption as an absolute value of the time course changes. (B) ABC values of the chemicals tested. The names of the developmental toxicants are written within a box. The toxicants causing limb malformation are labeled with gray color. Data are presented as the mean ± S.D. (n = 3)

To express the degree of signal disruption dynamics with a single parameter, the area between the curves (ABC) was calculated by temporally integrating the differences in the normalized luminescence between the vehicle control group and the chemical exposure group for each concentration condition (Figure 3A). Note that the positive and negative impacts on the luminescence intensity of each chemical are shown in Figure 3B with white and black bar graphs, respectively. As expected, there were concentration-dependent increases in the ABC value for the developmental toxicants and, in contrast, small changes were observed for the non-developmental toxicants, with a few exceptions. The ABC values of 2 out of 12 developmental toxicants tested, 6-aminonicotinamide (6-AN) and 5,5-dimethyl-2,4-oxazolidinedione (DMO), were almost independent on the concentration. Of the six non-developmental toxicants, ABC values of AcA and DHM showed relatively large, concentration-dependent increases (p < 0.001 for trend). Notably, for all seven toxicants causing limb malformation, ABC values were considerably increased in a concentration-dependent manner (p < 0.001 for trend), implying that the responsivity of the signal reporter cells to chemicals may be useful for detecting putative limb malformation toxicants. Interestingly, the limb malformation toxicants caused a uniformly positive or negative impact on the luminescence intensity in the course of the exposure at nearly all doses (Figure 3B). In contrast, the non-developmental toxicants AcA and DHM, which showed concentration-dependent increases in ABC, had both positive and negative effects on the luminescence, depending on the time of the exposure. Considering the direction of impact may provide an approach to eliminate false-positive toxicity signals, but further studies will be necessary to obtain more datasets.

### Area between the curves as a useful indicator for determining developmental toxicants

**Figure 4:**
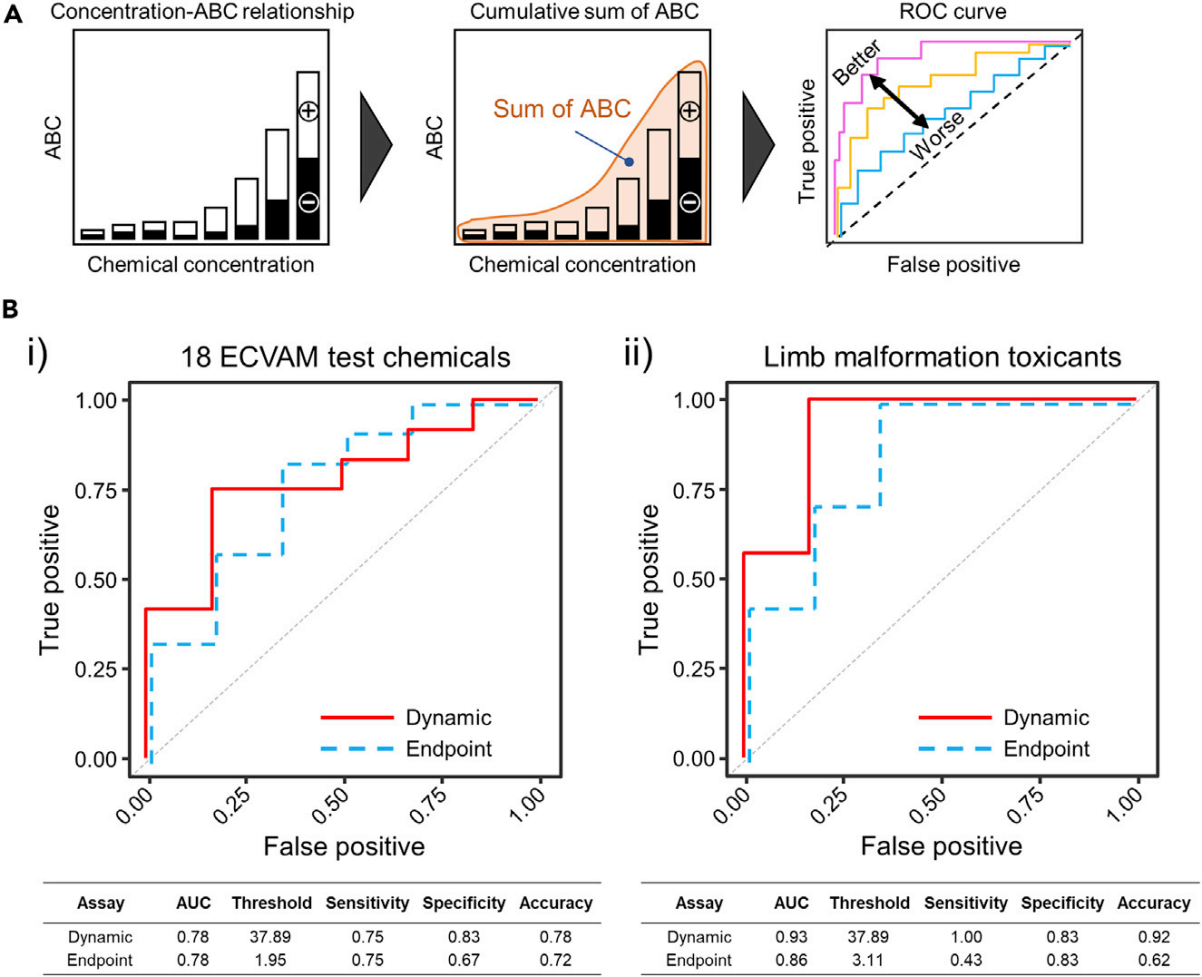
Receiver operating characteristic (ROC) curve analysis (A) Schematics of ROC curve calculation using ABC values. The sum of ABC values was calculated by adding individual ABC values obtained at each concentration for each chemical and used for ROC curve plotting. (B) ROC curves for the 18 ECVAM chemicals tested (i) and for toxicants causing limb malformation (ii). For comparison, the same calculations were performed for the single endpoint assay, in which the difference in the luminescence intensity at 24 h between the vehicle control group and chemical exposure group was measured instead of the ABC. The tables at the bottom summarize the performance of the dynamic and single endpoint assays

The receiver operating characteristic (ROC) curve is a plot that depicts the trade-off between the sensitivity and specificity across a series of cutoff points, which is an effective method for assessing the performance of a predictive test. The area under the ROC curve (AUC) provides a quantitative measurement of binary classification performance. We calculated the cumulative sum of ABC values and plotted the ROC curve (Figure 4A). To compare the performance of the dynamic assay with the single endpoint assay, the same calculations were conducted for the single endpoint assay and instead of the ABC, the difference in the luminescence intensity at 24 h between the vehicle control group and the chemical exposure group was used. When all 18 chemicals were classified as either developmental or non-developmental toxicants, the AUC was 0.78 for both the dynamic and single endpoint assays (Figure 4B(i)). Because the FGF signaling pathway is deeply involved in limb development, the same calculations were conducted for seven limb malformation toxicants and six non-developmental toxicants, excluding five non-limb malformation toxicants. In this classification, the AUC was 0.93 and 0.86 for the dynamic and single endpoint assays, respectively (Figure 4B(ii)). In general, the AUC between 0.5 and 0.7 indicates quite a low classification performance, whereas those between 0.7 and 0.9, and over 0.9 are considered to reflect good and excellent performance, respectively (Swets, 1988). Therefore, the results show that the dynamic reporter assay for limb malformation toxicants provided excellent classification performance.

## Discussion

In this study, we proposed an *in vitro* developmental toxicity assay based on the disruption of the FGF signaling pathway. Several studies have previously reported the use of signaling disruption assays to screen for the developmental toxicity of various chemicals using mouse embryonic stem cells that contain BMP and Wnt signal reporter genes (Kugler et al., 2015; Uibel et al., 2010; Uibel and Schwarz, 2015). However, in those assays, reporter cells were collected at a fixed time point to extract luciferase. Such single endpoint assay may be inappropriate for estimating signaling disruption because the extent of disruption changes in time (Purvis and Lahav, 2013). We also observed that following the exposure to chemicals, luminescence intensity dynamically changed, even after its normalization by the signal from solvent control (Figures 2C and 2D). Indeed, the single endpoint assay after the 24 h exposure provided a false negative estimation of the developmental toxicity for four out of seven toxicants causing limb malformation, namely ATRA, hydroxyurea, MeHg, and sodium salicylate, resulting in low sensitivity (Figure 4B(ii)). To obtain more reliable outcomes, we used the multiple time point, dynamic assay and integrated the extent of signaling disruption over time for each concentration of the chemical to obtain an ABC value (Figure 3A). We further summed all ABC values and plotted the ROC curve (Figure 4A). For all 18 chemicals, there was no difference in the AUC value (both were 0.78) between the dynamic and single endpoint assays, which was not advantageous over the outcomes of the previous studies (Luz and Tokar, 2018). However, for the seven toxicants known to cause limb malformations, the dynamic assay showed significantly improved performance (AUC = 0.93) compared with that of the single endpoint assay (AUC = 0.86). The particular applicability of the dynamic assay to the detection of malformation-causing substances is likely explained by the fact that the FGF signaling pathway is closely associated with limb malformation.

From the ROC curve for the dynamic assay in Figure 4B (i, ii), the threshold was determined by calculating the closest point to the upper left corner (the point at true positive 1.0 and false-positive 0), which provided the best estimation of developmental toxicants and non-toxicants. Table 2 summarizes the estimation, again indicating that our approach precisely determined the seven toxicants causing limb malformation. An interesting finding in this study was that different limb malformation toxicants had either uniformly positive or uniformly negative impact on the FGF/SRF signaling pathway (Figure 3B). During early embryogenesis and organogenesis, the FGF signaling pathway is strictly regulated by a number of components that provide feedback to balance up- and down-regulation (Böttcher and Niehrs, 2005; Carter et al., 2015). Our results suggest that chemicals exerting an irreversible impact on the feedback system in either direction may be developmental toxicants that cause limb malformations.

**Table 2:** Developmental (P: positive) and non-developmental (N: negative) toxicants as characterized by animal experiments and *in vitro* reporter assay

Chemical tested	Animal study (previous report^a^)	*In vitro* study (this study)	Sum of ABC values	Hedge's g (Sum of ABC vs. Threshold)
^†^ ATRA	P	P	65.85	1.15
^†^ HU	P	P	63.29	2.54
^†^ MAA	P	P	225.30	7.54
^†^ MeHg	P	P	77.73	1.24
^†^ MTX	P	P	80.48	1.90
^†^ SA	P	P	38.69	0.27
^†^ VPA	P	P	91.74	2.16
6-AN	P	N	24.88	−4.21
BA	P	N	24.18	−2.00
BrdU	P	P	83.91	2.27
DMO	P	P	50.29	0.34
LiCl	P	N	29.75	−2.60
AcA	N	N	37.09	−0.10
CAM	N	N	27.48	−1.75
DHM	N	P	68.18	3.85
DMP	N	N	24.41	−3.10
PenG	N	N	22.12	−3.58
SAC	N	N	33.99	−1.92

a Based on the developmental toxicity classification of chemicals used in a previous report (Genschow et al., 2002), the tested chemicals were categorized into positive (P) developmental toxicants and negative (N) (non-developmental) toxicants. Daggers (†) indicate limb/digit developmental toxicants. In the column of Hedge’s g (sum of ABC values vs. threshold), the underlined values indicate effect sizes above 0.2, which are empirically considered to be associated with moderate and larger effects (Brydges, 2019).

In the screening of new chemicals with unknown developmental toxicity using our approach, a statistical analysis needs to be conducted to determine whether the sum of ABC values exceeds the threshold value. In this statistical analysis, the effect size measured by the Hedge's g statistic, which indicates the degree of difference between two values independent of the sample size, may be useful (Sullivan and Feinn, 2012). An effect size value of 0.2 or more would indicate that there is a small but important difference (Brydges, 2019). When developmental toxicity was determined for 18 test chemicals, each sum of ABC values for limb malformation-inducing chemicals exceeded the threshold Hedge's g value of 0.2 (Table 2). The determination of effect size by calculating Hedge’s g statistic could be a useful quantitative approach for the high-throughput screening of a large number of chemicals. To further examine our approach, we performed experiments with 12 additional chemicals, including thalidomide, listed in the International Council for the Harmonization of Technical Requirements for Pharmaceuticals for Human Use (ICH) S5 guideline (Kanno et al., 2022). Of note, thalidomide and its two derivatives, lenalidomide, and pomalidomide, were classified as limb malformation-related chemicals. Therefore, this approach could be a powerful method to detect potential developmental toxicity in human.

With regard to individual chemicals, MeHg is well recognized as a developmental neurotoxicity substance responsible for Minamata disease, which caused limb malformations in babies born around a Chisso Corporation chemical factory (European Chemicals Agency, 2017). MeHg has been shown to cause anencephaly and limb malformations in chick embryos (Gilani, 1975) and cleft palate and limb malformations in mouse embryos (Su and Okita, 1976). However, an *in vitro* assay based on the inhibition of cardiomyocyte differentiation from mouse ES cells resulted in a false negative result for MeHg (Genschow et al., 2002). A true positive result for MeHg was shown using a similar assay based on the inhibition of neural differentiation (Kobayashi et al., 2017). In our study, MeHg induced concentration-dependent disruption of the FGF signaling pathway and was clearly determined to be a developmental toxicant. These findings support our hypothesis that the disruption of signaling pathways could be a more comprehensive marker for the assay of developmental toxicants compared to the differentiation inhibition to specific tissues. 5-bromo-2′-deoxyuridine (BrdU) is a developmental toxicant according to the ECVAM validation study, and it was classified as a non-limb malformation toxicant in this study based on the results of PubMed database mining (Table S1). We found only 7 hits for BrdU in 695 limb malformation-related articles, whereas our classification criteria required more than 10 hits for a limb malformation toxicant. However, BrdU aliases, such as 5′-bromo-2′-deoxyuridine, 5-bromodeoxyuridine, BudR, and bromodeoxyuridine, had an additional 10 hits in total. Furthermore, BrdU has been reported to cause polydactyly in mouse fetuses (Nakamura et al., 2000). These facts may explain why BrdU showed a concentration-dependent increase in the extent of FGF signaling disruption, similar to that afforded by the established limb malformation chemicals (Figure 3B). In contrast, 6-AN was also classified as a non-limb malformation toxicant and its aliases had a total of 17 hits in the articles on limb malformation. Additionally, 6-AN was shown to cause limb malformation in chick embryos (Honda et al., 1982). However, a considerable disruption of FGF signaling was not observed in our assay (Figure 3B). A previous study reported that 6-AN induced limb malformation by inhibiting the biosynthesis of glycosaminoglycans and proteoglycans (Honda et al., 1982). The mechanism responsible for 6-AN-induced malformation may not involve FGF signaling disruption, and this is probably why 6-AN was not classified as a developmental toxicant by our assay. Given that 6-AN and BrdU were chemicals with a potential to cause limb malformations, the ROC curve was replotted (Figure S6). Although the accuracy slightly decreased compared with that when it was automatically categorized by PubMed database mining (Figure 4B), the value (AUC = 0.87) was still considered to be relatively high. The non-developmental toxicants AcA and DHM caused concentration-dependent FGF signaling disruption, and DHM was determined to be a false-positive call. Notably, DHM was also reported to evoke a false-positive signal in several previous *in vitro* developmental toxicity studies (Genschow et al., 2002; Suzuki et al., 2011). Epidemiological studies have concluded that there is no statistically significant association between DHM and congenital malformations. However, in a small number of cases, DHM exposure has been reported to cause congenital malformations such as cleft lip, cleft palate, and lateral limb defects in fetuses (Gilboa et al., 2009). Although statistical causality has not been proven in epidemiological studies, many *in vitro* developmental toxicity studies have judged it to be a developmental toxicant. Therefore, more detailed toxicological information is required to establish whether DHM causes any adverse effects on the fetus.

In conclusion, we have established a developmental toxicity assay focused on the FGF-activated RTK/SRF signaling pathway and enhanced its performance by integrating dynamic signal disruption (ABC). The assay afforded a good prediction of developmental toxicants (AUC = 0.78) and had excellent sensitivity to detect limb malformation chemicals (AUC = 0.93). Further work is required to cross-validate the assay by using additional compounds as well as to combine it with reporter assays focused on other types of signaling.

### Limitations of the study

Our assay focused on the FGF/SRF signaling pathway and generated particularly good estimations when it was applied to toxicants inducing limb malformation. However, it is obvious that further studies are needed to test this assay by cross-validation approaches with other chemicals that are known to be either developmental or non-developmental toxicants in animals and/or humans. Several other major signaling pathways are associated with developmental morphogenesis, such as Wnt/β-catenin, BMP/SMAD, and Hedgehog/GLI (Sanz-Ezquerro et al., 2017). Combinations of reporter cells that report the activity of these signaling pathways may provide a more comprehensive assessment of developmental toxicants. In addition, human iPSCs were used as reporter cells in this study. Recent advances in iPSC research allow the induction of iPSC differentiation into specific cell types, and *in vitro* developmental toxicity assays have been performed using human iPSC-derived cells differentiated into embryoid bodies (Jaklin et al., 2020) and cardiomyocytes (Hoang et al., 2021). Therefore, a set of reporter cells reflecting the activity of different signaling pathways at distinct differentiation stages, for example, undifferentiated or triploblastic (endoderm/mesoderm/ectoderm) cells, will lead to further development of robust and versatile screening approaches. Because of the inevitable differences between *in vivo* and *in vitro* settings, this assay is useful only for the initial screening of chemicals and it has to be combined with other assays, including animal experiments, to provide definitive conclusions about the teratogenic potential of the tested compounds.

## STAR★Methods

### Key resources table

**Table:** 

REAGENT or RESOURCE	SOURCE	IDENTIFIER
**Antibodies**		

Human Alkaline Phosphatase/ALPL Antibody	R&D Systems	Cat#MAB1448-SP; RRID:AB_2258295
Donkey Anti-Goat IgG H&L (Alexa Fluor 555)	Abcam	Cat#ab150130
Goat Anti-Mouse IgG H&L (Alexa Fluor 488)	Abcam	Cat#ab150113; RRID:AB_2576208
Human/Mouse Oct-3/4 Antibody	R&D Systems	Cat#AF1759-SP; RRID:AB_354975

**Bacterial and virus strains**		

*E. coli* HST08 Premium Competent Cells	Takara Bio	Cat#SD1423
pMK232 (CMV-OsTIR1-PURO)	Natsume et al. (2016)	Addgene (Plasmid #72834)
pNL (NlucP/SRE/Hygro)	Promega	Cat#CS177601

**Chemicals, peptides, and recombinant proteins**		

Alt-R® CRISPR-Cas9 crRNA, 2 nmol	Integrated DNA Technologies (Richardson et al., 2016)	N/A
Alt-R® CRISPR-Cas9 tracrRNA 5 nmol	Integrated DNA Technologies	Cat#1072532
Alt-R® S.p. HiFi Cas9 Nuclease V3, 100 μg	Integrated DNA Technologies	Cat#1081060
Neon™ Transfection System 10 μL Kit	Thermo Fisher	Cat#MPK1025
Geltrex™ LDEV-Free Reduced Growth Factor Basement Membrane Matrix	Thermo Fisher	Cat#A1413202
StemFlex™ Medium	Thermo Fisher	Cat#A3349401
TrypLE™ Select Enzyme (1X), no phenol red	Thermo Fisher	Cat#12563011
Dimethyl Sulfoxide	Merck	Cat#D4540; CAS: 67-68-5
CultureSure® Y-27632	FUJIFILM Wako Pure Chemical	Cat#030-24021; CAS: 331752-47-7
Puromycin Dihydrochloride	Thermo Fisher	Cat#A1113803
STEMdiff APEL2 Medium	Veritas	Cat#ST-05275
Recombinant Human EGF	PeproTech	Cat#AF-100-15
Heat Stable Recombinant Human bFGF	Life Technologies	Cat#PHG0367V
Human BMP-4 recombinant protein	Proteintech	Cat#HZ-1045
Recombinant Human TGF-β3	PeproTech	Cat#AF-100-36E-10UG
Recombinant Human Wnt-3a Protein	R&D Systems	Cat#5036-WN-010/CF
PD0325901	FUJIFILM Wako Pure Chemical	Cat#162-25291; CAS: 391210-10-9
CCG-203971	Cayman Chemical	Cat#15075; CAS: 1443437-74-8
all-*trans*-Retinoic acid	Merck	Cat#PHR1187; CAS: 302-79-4
Hydroxyurea	Merck	Cat#H8627; CAS: 127-07-1
Methoxyacetic acid	Merck	Cat#194557; CAS: 625-45-6
Methylmercury chloride	Merck	Cat#33368; CAS: 115-09-3
Methotrexate hydrate	Merck	Cat#A6770; CAS: 133073-73-1
Sodium salicylate	Merck	Cat#28-4040; CAS: 54-21-7
Valproic acid	FUJIFILM Wako Pure Chemical	Cat#225-01072; CAS: 99-66-1
6-Aminonicotinamide	Merck	Cat#A68203; CAS: 329-89-5
Boric acid	FUJIFILM Wako Pure Chemical	Cat#021-02195; CAS: 10043-35-3
5-Bromo-2′-deoxyuridine	Merck	Cat#B5002; CAS: 59-14-3
5,5-Dimethyl-2,4-oxazolidinedione	Merck	Cat#D7631; CAS: 695-53-4
Lithium chloride	NACALAI TESQUE	Cat#09887-82; CAS: 7447-41-8
Acrylamide	Merck	Cat#A9099; CAS: 79-06-1
D-Camphor	Merck	Cat#PHR1119; CAS: 464-49-3
Diphenhydramine hydrochloride	Merck	Cat#D3630; CAS: 147-24-0
Dimethyl phthalate	Tokyo Chemical Industry	Cat#P0302; CAS: 131-11-3
Penicillin G sodium salt	Merck	Cat#PENNA; CAS: 69-57-8
Sodium saccharin	Merck	Cat#47839; CAS: 82385-42-0

**Critical commercial assays**		

KAPA SYBR® FAST qPCR Master Mix (2x)	NIPPON Genetics	Cat#KK4602
Cell Counting Kit-8	Dojindo	Cat#CK04
Nano-Glo® Luciferase Assay System	Promega	Cat#N1110
Nano-Glo® Endurazine™ Substrate	Promega	Cat#N2571

**Experimental models: cell lines**		

Human Induced Pluripotent Stem (iPS) Cells	Riken BRC Cell Bank	201B7 (RCB Cat# HPS0063, RRID: CVCL_A324)

**Oligonucleotides**		

Primer: *AAVS1* Forward: GGACCACTTTGAG CTCTACTGGCTTCTGCG	This manuscript	N/A
Primer: *AAVS1* Reverse: GCTGTCCTGGGCA AACAGCATAAGCTGGTCAC	This manuscript	N/A
Primer: *EGR1* Forward: CCCTACGAGCACC TGACCGC	This manuscript	N/A
Primer: *EGR1* Reverse: GTCTCCACCAGCAC CTTCTCG	This manuscript	N/A
Primer: *FOS* Forward: GGGCTGGCGTTGTG AAGAC	This manuscript	N/A
Primer: *FOS* Reverse: AGTTGGTCTGTCTCC GCTTGGA	This manuscript	N/A
Primer: *SRRY4* Forward: TCAGGATTTACACA GACGTGGG	This manuscript	N/A
Primer: *SPRY4* Reverse: GCAAACCGCTCAA TACAGGC	This manuscript	N/A
Primer: *GAPDH* Forward: TGACTTCAACAG CGACACCC	This manuscript	N/A
Primer: *GAPDH* Reverse: GCCAAATTCGTT GTCATACCAGG	This manuscript	N/A

**Recombinant DNA**		

Donor plasmid for gene editing	This manuscript	N/A

**Software and algorithms**		

R (version 4.0.3)	R Core Team	https://cran.r-project.org/bin/windows/base/
SPSS software	IBM	Ibm.com/spss/statistics
All original code	This manuscript	Mendeley Data: https://doi.org/10.17632/79nmyyv99z.2

### Resource availability

#### Lead contact

Further information and requests for resources and reagents should be directed to and will be fulfilled by the lead contact, Junji Fukuda (fukuda@ynu.ac.jp)

#### Materials availability

This study did not generate new unique reagents.

### Experimental models and subjects

#### Cell lines and culture conditions

Human iPSC line 201B7 (Riken Cell Bank, Japan) was routinely cultured in the StemFlex medium (A3349401, Thermo Fisher Scientific, Waltham, MA, USA) on culture plates coated with Geltrex Matrix (A1413202, Thermo Fisher Scientific). When cells reached 60%–80% confluence, the culture medium was removed, and the cells were rinsed with PBS and incubated with TrypLE Select (12563011, Thermo Fisher Scientific) for 4–5 min at 37°C. Then, the triple amount of culture medium was added to the suspension. Cells were then pelleted by centrifugation (200 × g for 4 min), resuspended in the culture medium containing 10 μM ROCK inhibitor Y-27632 (CS-0131, ChemScene, South Brunswick Township, NJ, USA), and seeded onto new Geltrex-coated culture plates at a viable cell density of 1.25 × 10^4^ cells/cm^2^. The cells were incubated at 37°C in a humidified chamber containing 5% CO_2_.

#### Development of stable transgenic iPSCs

Custom Alt-R CRISPR-Cas9 crRNA and generic tracrRNA (1072532, Integrated DNA Technologies, IA, USA) were resuspended to 200 μM in a Tris-EDTA buffer solution (pH 8.0) (06890-54, Nacalai Tesque, Kyoto, Japan). Next, crRNA:tracrRNA complexes (gRNA) were generated by incubating equimolar ratios at 95°C for 5 min and then returning to room temperature. The custom crRNA sequence was 5′-acagtggggccactagggac-3′designed with reference to previous studies (Richardson et al., 2016). To form Cas9 RNP complexes, a mixture of gRNA and Alt-R S.p. HiFi Cas9 Nuclease V3 (1081060, Integrated DNA Technologies) was incubated for 15 min at room temperature. After complex formation, 1 × 10^5^ human iPSCs were transfected using the Neon Transfection System 10 μL kit (MPK1025, Thermo Fisher Scientific) with the following electroporation settings: 1,200 V; 20 ms; 2 pulses. Subsequently, the cells were seeded onto new Geltrex-coated 24-well culture plates. In 48 h after electroporation, 0.5% puromycin (A1113803, Thermo Fisher Scientific) was added to the culture medium. After 9 days of puromycin selection, the expanded cell colonies were isolated and cultured on 6-well culture plates.

### Methods details

#### Chemicals tested

The tested chemicals (Table 1) were selected from the list of the ECVAM International Validation Study on *in vitro* embryotoxicity tests (Genschow et al., 2002). Because it has been reported that FGF signaling pathway contributes to limb formation during fetal mouse organogenesis (Ornitz and Itoh, 2015), the chemicals involved in limb malformation were selected from the list of ECVAM chemicals based on database search results. The keywords "(limb OR digit) AND (teratogen OR developmental toxicity) AND (pregnancy OR fetal OR fetus OR birth OR infant)" were input into PubMed, and hit articles with available abstracts were extracted. The sentences in the abstract were divided into individual words by using natural language processing that included morphological analysis (supplemental information), and the words related to chemical substances were extracted from them by matching with PubChem and ChemSpider searches. ECVAM chemicals with more than 10 occurrences of related words were classified as limb malformation toxicants.

All chemicals were dissolved in the appropriate vehicle, either phosphate buffered saline (PBS, 70013032, Thermo Fisher Scientific), which was the vehicle of the first choice, or dimethyl sulfoxide (DMSO, D4540-100ML, Sigma-Aldrich, St. Louis, MO, United States), according to the ECVAM study (Genschow et al., 2004).

#### Construction of plasmids

All donor plasmids used in this study were generated using the In-Fusion® HD Cloning Kit (639633, Takara Bio, Shiga, Japan). pMK232 (CMV-OsTIR1-PURO) was a gift from Masato Kanemaki (Addgene plasmid # 72834; http://n2t.net/addgene:72834; RRID:Addgene_72834). The DNA sequences encoding the nanoluciferase (*Nluc*) reporter gene downstream of the SRE and *PuroR* gene were amplified from the pNL[NlucP/SRE/Hygro] Vector (Promega, Madison, WI, USA) and inserted into bacterial plasmid pMK232 with adeno-associated virus integration site 1 (*AAVS1*) homology arms of about 800 bp at both ends of the donor plasmid for efficient knock-in. The integration of the construct was confirmed by Sanger sequencing (Eurofins Genomics K.K.). Plasmids were propagated by using *E. coli* Cells (SD1423, Takara Bio) and were purified using the Plasmid DNA Extraction Mini Kit (FAPDE001, Favorgen Biotech, Ping-Tung, Taiwan).

#### Genomic DNA extraction, PCR, and DNA sequence

When selected colonies grew to 60%–80% confluence, genomic DNA was extracted from the cells using TRIzol Reagent (15596026, Thermo Fisher Scientific) according to the manufacturer’s protocol. Genomic regions containing SRE, *Nluc*, and *PuroR* were PCR amplified using the following primers: forward, 5ʹ-ggccggttaatgtggctctggttctgggtac-3ʹ; reverse, 5ʹ-cccaggatcctctctggctccatcgtaagc-3ʹ. PCR products of selected cells were sent for Sanger sequencing and BLAST searched against the human genome and donor plasmid to confirm DNA insertion.

#### Total RNA extraction and RT-qPCR

Total RNA was extracted from cells using the RNeasy Mini Kit (74106, Qiagen, Hilden, Germany) according to the manufacturer’s protocol. Reverse transcription (RT) of RNA to generate complementary DNA (cDNA) was performed in a total volume of 20 μL by using FastGene cDNA Synthesis 5× ReadyMix with random hexamer primers for mRNA (NE-LS64, Nippon Genetics, Tokyo, Japan) according to the manufacturer’s instructions.

After mixing the cDNA with the KAPA SYBR Fast qPCR kit (KK4602, Kapa Biosystems, Wilmington, MA, USA), 10 μL of the mixture containing cDNA synthesized from 5 ng total RNA was dispensed into each well of the PCR plate. Real-time PCR was performed on a LightCycler 96 System (05815916001, Roche Diagnostics, Basel, Switzerland) using the following cycling parameters: 3 min at 95°C (heat activation step); 40 cycles of 10 s at 95°C, 20 s at 60°C, and 1 s at 72°C. Dissociation curve analyses were performed using the default settings of the instrument immediately after each PCR run. For each unknown sample, the relative amount was calculated using linear regression analysis from the relative standard curves. The relative target gene mRNA expression value was then obtained by dividing the target gene expression by the value for *GAPDH* mRNA expression as internal reference.

#### Immunocytochemistry

For immunocytochemistry, human iPSCs were seeded at a viable cell density of 1.0 × 10^4^ cells/well onto Geltrex-coated flat-bottom 96-well black culture plates (6005182, PerkinElmer) in the culture medium containing 10 μM ROCK inhibitor Y-27632. At 24 h after seeding, the medium was replaced with the StemFlex culture medium. After reaching 30%–40% confluence, the medium was discarded, and the cells were fixed with 4% paraformaldehyde (168-23255, Fujifilm Wako Pure Chemical, Tokyo, Japan) in PBS for 10 min at room temperature. Following fixation, the fixative was discarded, and the cells were washed with PBS three times for 5 min with gentle rocking. Cell membranes were permeabilized with 0.2% Triton-X100 (X100-5ML, Sigma-Aldrich) in PBS for 10 min at room temperature and washed with PBS. Blocking buffer containing 1% bovine serum albumin (013-23291, Fujifilm Wako Pure Chemical), 22.52 mg/mL glycine (50046-50G, Sigma-Aldrich), and 0.1% Tween 20 (T0543, Tokyo Chemical Industry, Tokyo, Japan) in PBS was added, and the cells were incubated for 30 min at room temperature. The blocking buffer was discarded prior to the antibody staining. The cells were then incubated with primary antibodies overnight at 4°C. The next day, the primary antibody solutions were discarded, and the plates were washed three times with PBS for 5 min with gentle rocking. The appropriate labelled secondary antibodies were diluted in a blocking buffer, applied to the cells, and then incubated for 1 h at room temperature. Then, the secondary antibody solutions were discarded, and the cells were washed three times with PBS for 5 min with gentle rocking. The cells were counterstained with DAPI (D9542, Sigma-Aldrich). Visualization of the antigen-antibody complexes was performed using an all-in-one fluorescence microscope (BZ-X710, Keyence, Osaka, Japan), and images were acquired using a BZ-X Analyzer (Keyence).

#### Cell viability assay

For cell viability assays, human iPSCs were seeded at a viable cell density of 1.5 × 10^4^ cells/well onto Geltrex-coated flat-bottom 96-well culture plates. Sterilized water was added to the wells directly facing the outer borders of the plates. After reaching 70%–80% confluence, cells were incubated with serially diluted chemicals for 20 h, and cell viability was measured by adding 10 μL of the Cell Counting Kit-8 (CCK-8) kit solution (CK04, Dojindo Laboratories, Kumamoto, Japan) to each well. The cells were then incubated at 37°C and 5% CO_2_ for 4 h with CCK-8 solution, after which the absorbance of cellular supernatants was measured at 450 nm according to the manufacturer's protocol. In all tests, blanks were prepared from cell-free controls using the CCK-8 reagent. The blank absorbance values were subtracted from those of the sample wells. The absorbance readings obtained during the experimental conditions was compared with that of the vehicle control group to calculate cell viability as follows:

$$Cell\;viability=\frac{Abs_{sample}-Abs_{blank}}{Abs_{vehicle}-Abs_{blank}}$$

The cell viability assay data were further analyzed using scripts written in R (version 4.0.3). The concentration–response curves were fitted using a four-parameter log-logistic model described by Ritz et al. (2015), using R package drc' with its functions drm() and LL.4() according to the following equation (Ritz et al., 2015):

$$f\left(x,\left(b,c,d,e\right)\right)=c+\frac{d-c}{1+exp\left(b\left(\log\left(x\right)-\log\left(e\right)\right)\right)}$$

The half-maximal growth inhibitory concentration (IC_50_) of each chemical was estimated using the same equation and set as the maximum concentration in the signaling disruption assay. For the chemicals for which IC_50_ could not be estimated, the maximum concentration was set to the concentration at which no precipitation of the chemical was observed in the medium, with an upper limit of 1% for PBS and 0.1% for DMSO at each vehicle concentration according to a previous report (Nagahori et al., 2016).

#### Live-cell luciferase reporter assays

Cells were seeded at a viable cell density of 5.0 × 10^3^ cells/well onto Geltrex-coated 96-well white 1/2 area culture plates (6005760, PerkinElmer) in the culture medium containing 10 μM ROCK inhibitor Y-27632. Sterilized water was added to the wells directly facing the outer borders of the plates. After the cells reached >90% confluence, the medium was changed to the STEMdiff APEL2 medium (ST-05275, Veritas, Tokyo, Japan) for 24 h. Live-cell luminescence detection experiments were performed with the APEL2 medium supplemented with 1% NanoLuc substrate Nano-Glo® Endurazine (N2570, Promega). After the application of the substrate-containing medium, the cells were equilibrated for 2 h in a regular incubator before the exposure to chemicals.

Serially diluted chemicals were added to each well and then 2 ng/mL heat stable recombinant human bFGF (PHG0367, Thermo Fisher Scientific) was added 1 h later, and the cells were then incubated for 24 h. Raw luminescence intensity data were obtained using a plate reader (SpectraMax i3x, Molecular Devices, San Jose, CA, United States), and background luminescence was obtained from the vehicle-treated control group (PBS [+ 0.1% BSA] and vehicle for chemicals) for each time series. The luminescence during experimental conditions was compared to that of the vehicle-treated control group to calculate the relative light unit (RLU). After measuring luminescence at 24 h, cell viability was measured by adding 30 μL of the Cell Viability Assay Kit (ab112122, Abcam, Cambridge, United Kingdom) to each well. The cells were then incubated at 37°C and 5% CO_2_ for 3 h, after which the fluorescence intensity of each well was measured according to the manufacturer's protocol at the excitation and emission wavelengths of 490 and 535 nm, respectively. In all tests, blanks were prepared from cell-free controls using the Cell Viability Assay Kit reagent. Blank fluorescence intensities were subtracted from those of the sample wells. The fluorescence intensities were compared to those of the vehicle control group to calculate cell viability, as mentioned above.

#### Scaling and calculation of ABC

To adjust the scale of each experiment, RLU obtained from live-cell luciferase reporter assays were normalized by the min-max scaling method using the following equation:

$$x_{scaled}=\frac{x-x_{min}}{x_{max}-x_{min}}$$

Fold change values were calculated by comparing chemical-treated RLU to vehicle-treated RLU (bFGF and vehicle for chemicals) at the same time point. Smoothing spline regressions with six degrees of freedom was performed to model the relationship between log-scaled fold change values and time series utilizing R package 'mgcv' with its function gam() (Wood, 2018). The distance (h) between the spline curves of the vehicle control group (a) and the chemical exposure group (b) at a certain time point was calculated:

$$h_{i}=\left|a\left(t_{i}\right)-b\left(t_{i}\right)\right|$$

The areas between the two curves were calculated by integrating the areas of all regions divided by the time change Δt as a trapezoid.

$$ABC\approx \sum_{i}\left[\frac{h_{i+1}+h_{i}}{2}\right]\Delta t_{i}$$

#### ROC curve analysis

ROC curve analysis based on logistic regression models was performed to identify the optimal threshold value for prediction purposes, utilizing R package 'pROC' with its function coords(). The AUC, sensitivity, specificity, and accuracy values were reported for the optimal thresholds. The threshold was determined by calculating the closest point to the upper left corner of the ROC curve (the point at true positive 1.0 and false positive 0).

### Quantification and statistical analysis

Details regarding quantification and statistical analysis are provided in each figure legends. All data were shown as mean ± S.D. calculated from multiple independent experiments. The tests of the statistical significance of differences were performed using SPSS software. For time series data, two-way analysis of variance was performed initially, and multiple comparisons using the Bonferroni correction were performed for all groups when there was a significant treatment×time interaction. Effects were considered statistically significant if *P* < 0.05. To evaluate the concentration dependence of ABC for each chemical, the Jonckheere–Terpstra trend test was performed using R package 'PMCMRplus' with its function JonckheereTest(). Concentration dependence was considered statistically significant if *P* < 0.001.

## Data Availability

• This study did not generate a new dataset. All data reported in this paper will be shared by the lead contact upon request. • All original code has been deposited at Mendeley Data and is publicly available as of the date of publication. DOIs are listed in the key resources table. • Any additional information required to reanalyze the reported data is available from the lead contact upon request.

## Footnotes

Supplemental information can be found online at https://doi.org/10.1016/j.isci.2022.103770.
