# Supplementary material for: Establishment of a developmental toxicity assay based on human iPSC reporter to detect FGF signal disruption
Source: iScience. 2022 Jan 15;25(2):103770. doi: 10.1016/j.isci.2022.103770 (PMC8819105; doi:10.1016/j.isci.2022.103770)
Supplement: Document S1. Figures S1–S6 and Tables S1 and S2 [file mmc1.pdf]

iScience, Volume *25*

Supplemental information

Establishment of a developmental toxicity assay based on human iPSC reporter to detect FGF signal disruption

Seiya Kanno, Yusuke Okubo, Tatsuto Kageyama, Lei Yan, Satoshi Kitajima, and Junji Fukuda

Figure S1. Generation of RTK/SRF signal reporter cells from human iPSCs, Related to Figure 1.

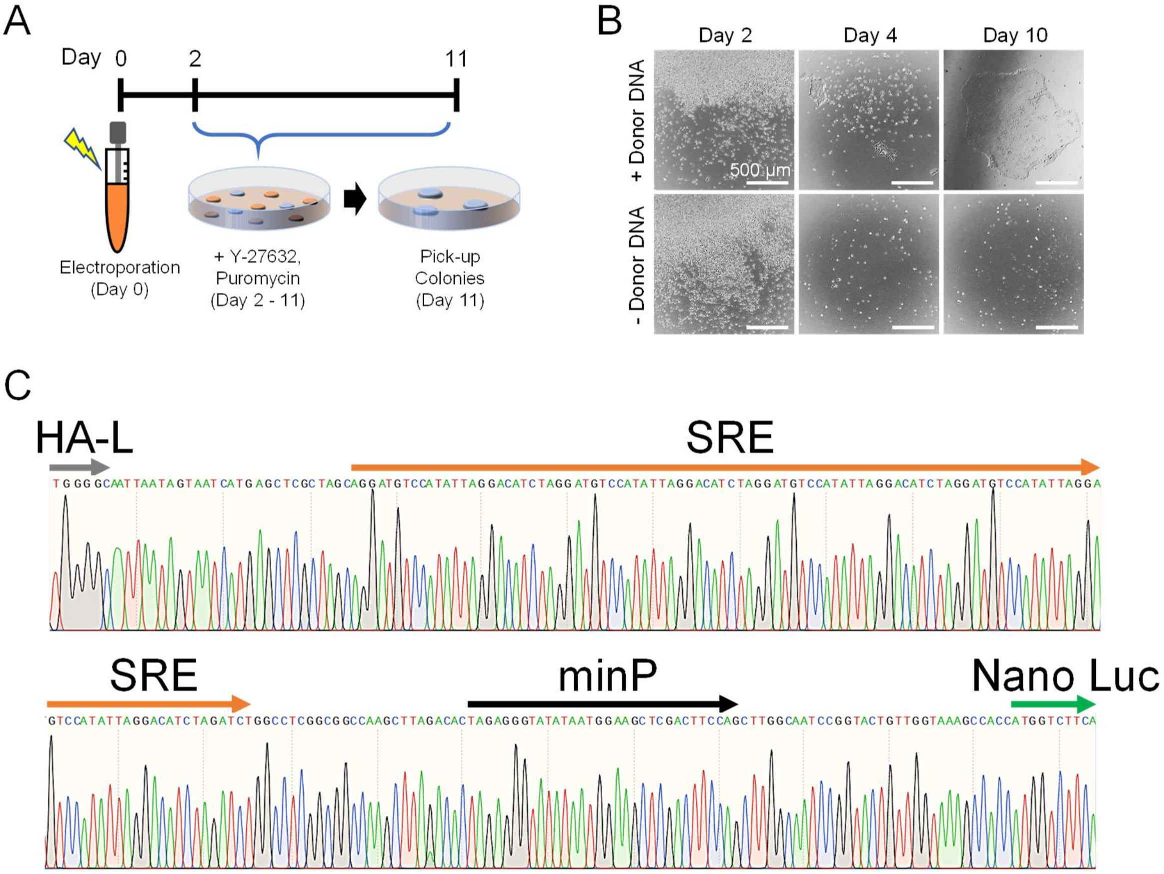

(A) Schematic procedures. Human iPSCs were electroporated with gRNA and Cas9 protein with or without donor DNA (day 0) and then treated with puromycin from day 2 to day 11. (B) Colony formation. Transfected cells grew and formed colonies, whereas non-transfected cells died in the presence of puromycin. (C) Representative Sanger sequencing chromatograms of the reporter cell line.

Figure S2. Cell survival assay to determine maximum concentration of chemicals, Related to Table 1.

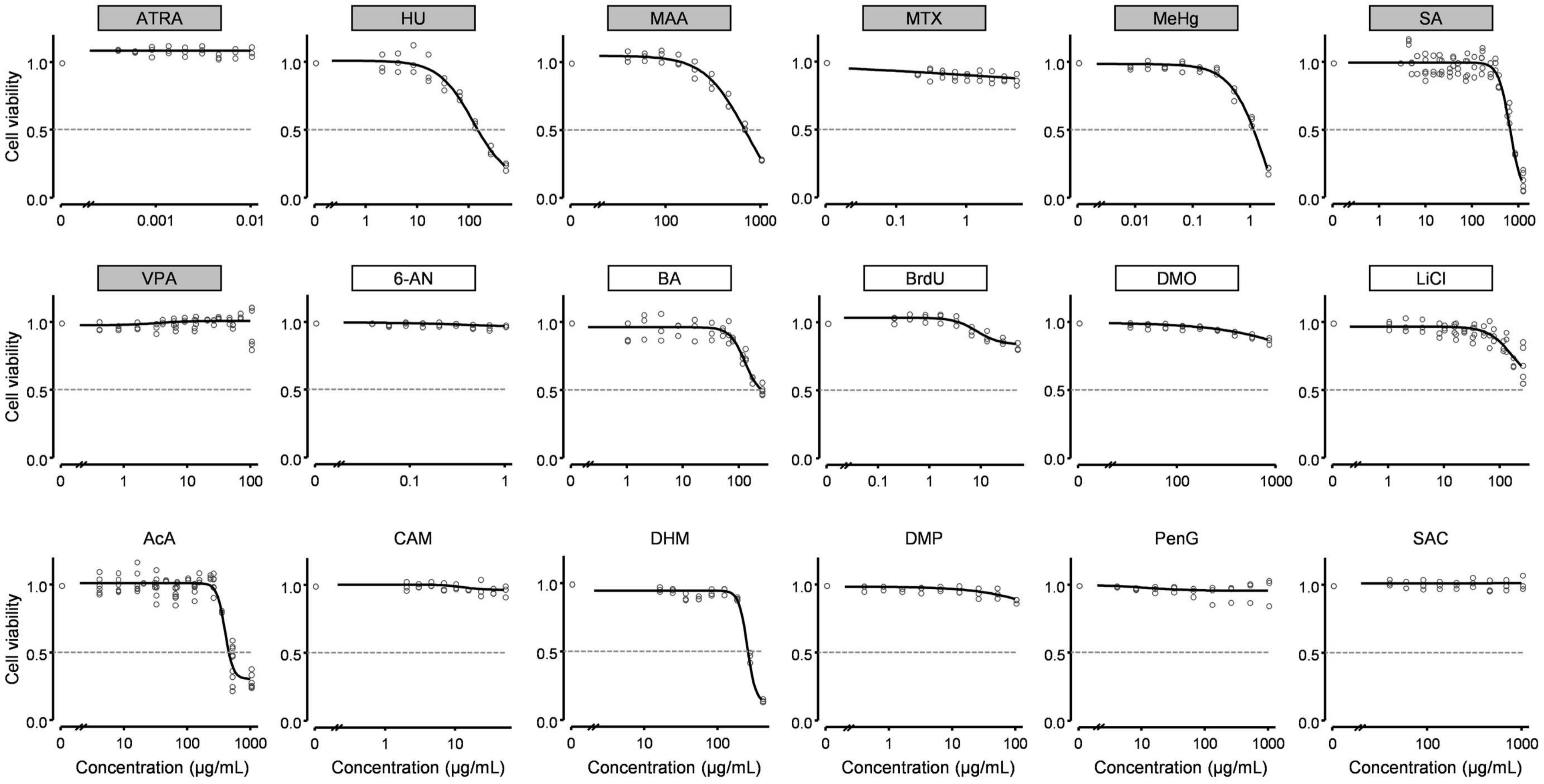

Human iPSCs were exposed to serially diluted chemicals, and cell viability was assessed by the CCK-8 assay at 24 h (n = 3). The lines represent four-parameter log-logistic regression function curves fitted to the experimental data. The maximum concentration was either the IC_{50} value determined from the fit or the maximum soluble concentration when the viability remained above 50%. The names of the developmental toxicants are written within a box. Toxicants causing limb malformation are labelled with gray color.

Figure S3. Changes in luminescence intensity induced by the developmental toxicants causing limb malformation tested in the live-cell luciferase assay, Related to Figure 2.

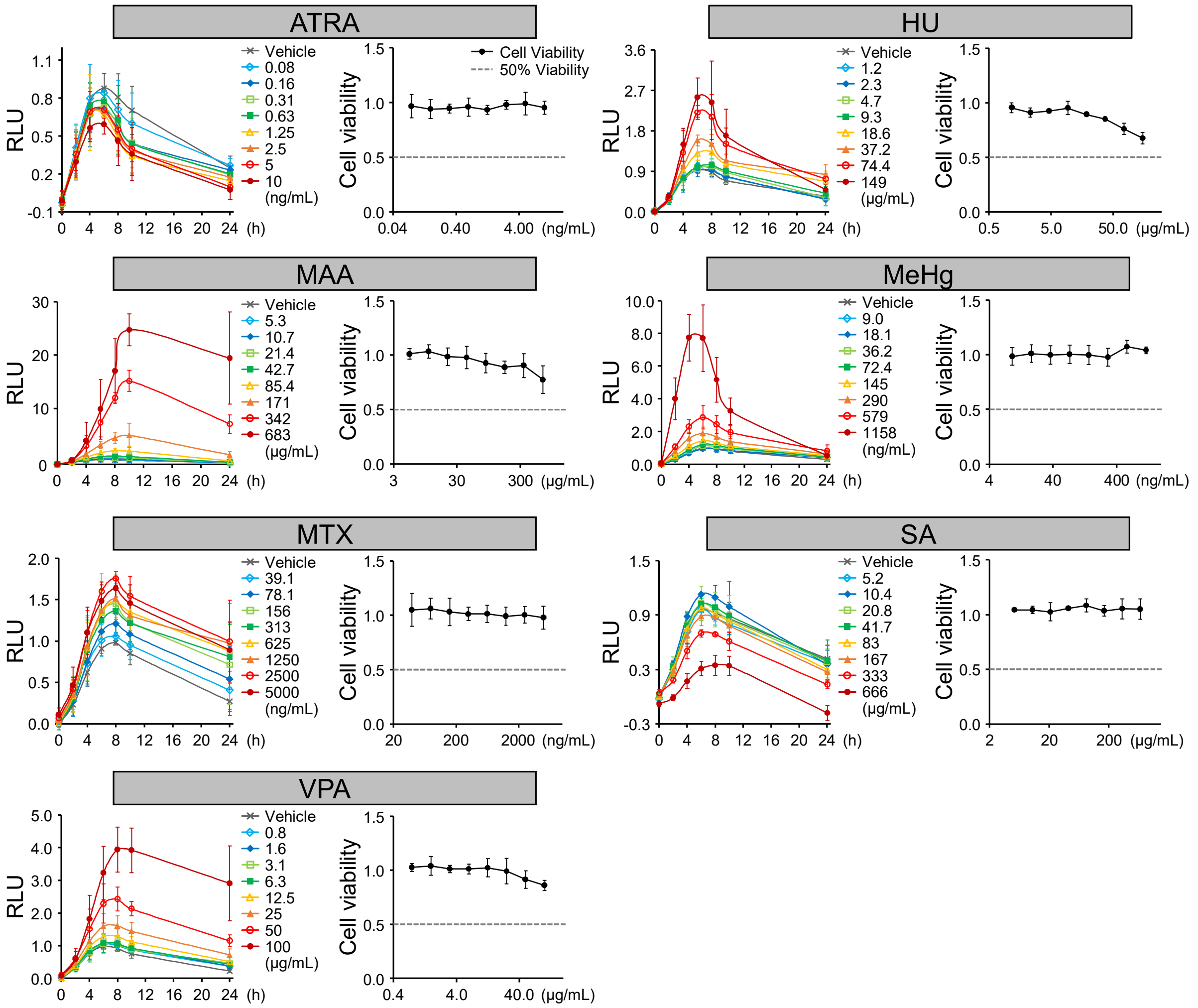

RTK/SRF signal reporter cells were exposed to the chemicals 1 h before (−1 h) the treatment with bFGF (0 h), and the luminescence intensity was monitored over 24 h (0–24 h). Cell viability was measured 3 h later (27 h). The intensity was normalized to that of the vehicle control at each time point. Further normalization was conducted using the min-max scaling method, where the minimum and maximum in the vehicle control with FGF stimulation were considered to be 0 and 1, respectively, and expressed as relative light units (RLU). Cell viability was normalized to that of vehicle control. Data represent the mean ± S.D. calculated from three independent experiments. The names of the developmental toxicants are written within a box. Toxicants causing limb malformation are labelled with gray color.

Figure S4. Changes in luminescence intensity induced by the developmental toxicants tested in the live-cell luciferase assay, Related to Figure 2.

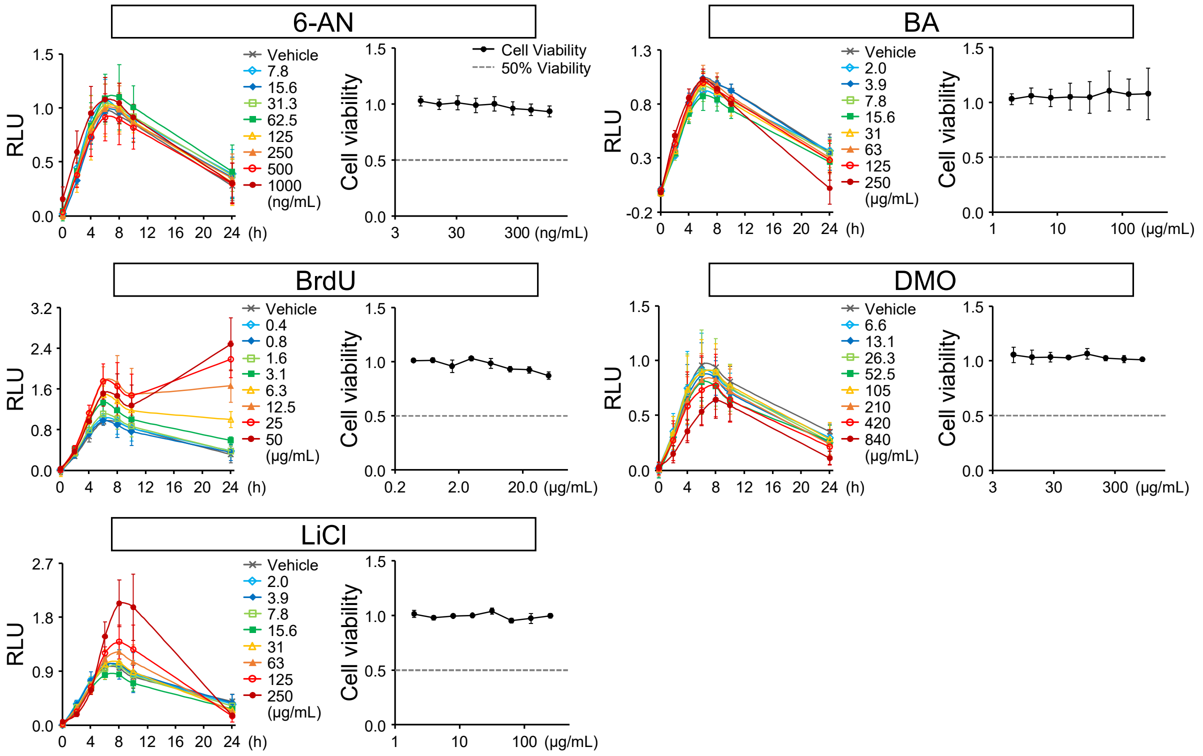

RTK/SRF signal reporter cells were exposed to the chemicals 1 h before (−1 h) the treatment with bFGF (0 h), and the luminescence intensity was monitored over 24 h (0–24 h). Cell viability was measured 3 h later (27 h). The intensity was normalized to that of the vehicle control at each time point. Further normalization was conducted using the min-max scaling method, where the minimum and maximum in the vehicle control with FGF stimulation were considered to be 0 and 1, respectively, and expressed as relative light units (RLU). Cell viability was normalized to that of vehicle control. Data represent the mean ± S.D. calculated from three independent experiments. The names of the developmental toxicants are written within a box.

Figure S5. Changes in luminescence intensity induced by the non-developmental toxicants tested in the live-cell luciferase assay, Related to Figure 2.

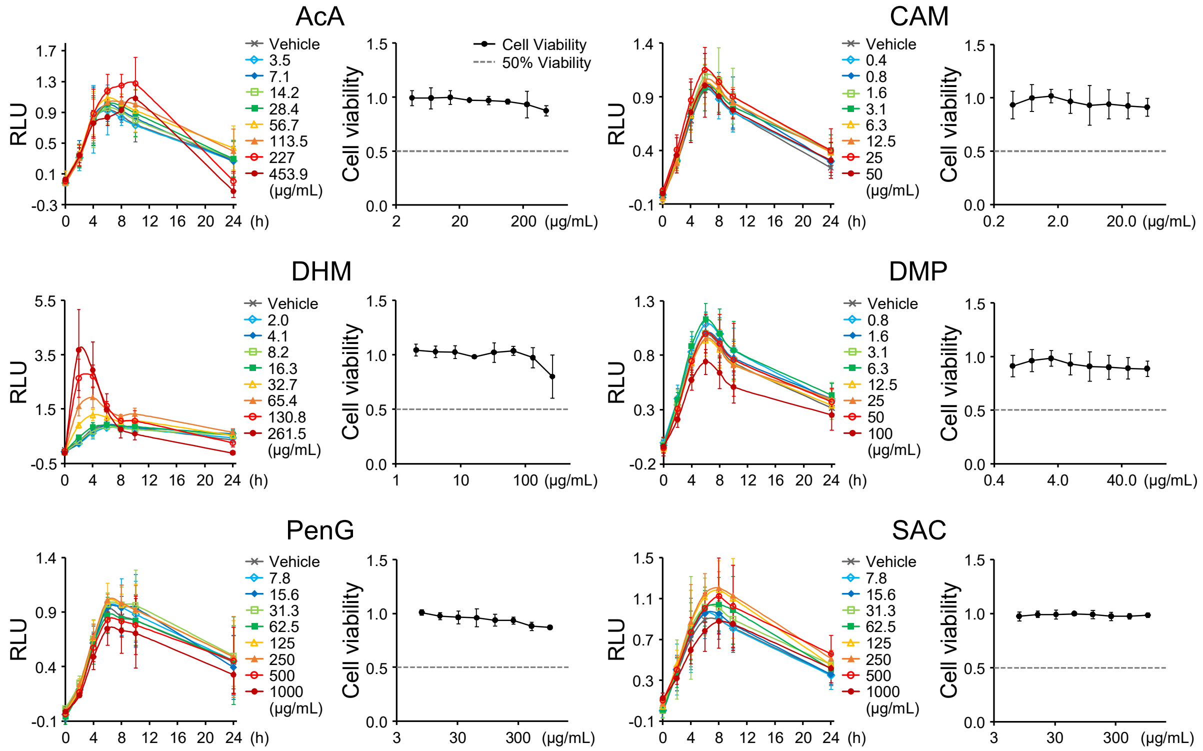

RTK/SRF signal reporter cells were exposed to the chemicals 1 h before (−1 h) the treatment with bFGF (0 h), and the luminescence intensity was monitored over 24 h (0–24 h). Cell viability was measured 3 h later (27 h). The intensity was normalized to that of the vehicle control at each time point. Further normalization was conducted using the min-max scaling method, where the minimum and maximum in the vehicle control with FGF stimulation were considered to be 0 and 1, respectively, and expressed as relative light units (RLU). Cell viability was normalized to that of vehicle control. Data represent the mean ± S.D. calculated from three independent experiments.

Figure S6. ROC curve analysis assuming 6AN and BrdU are limb malformation chemicals, Related to Figure 4.

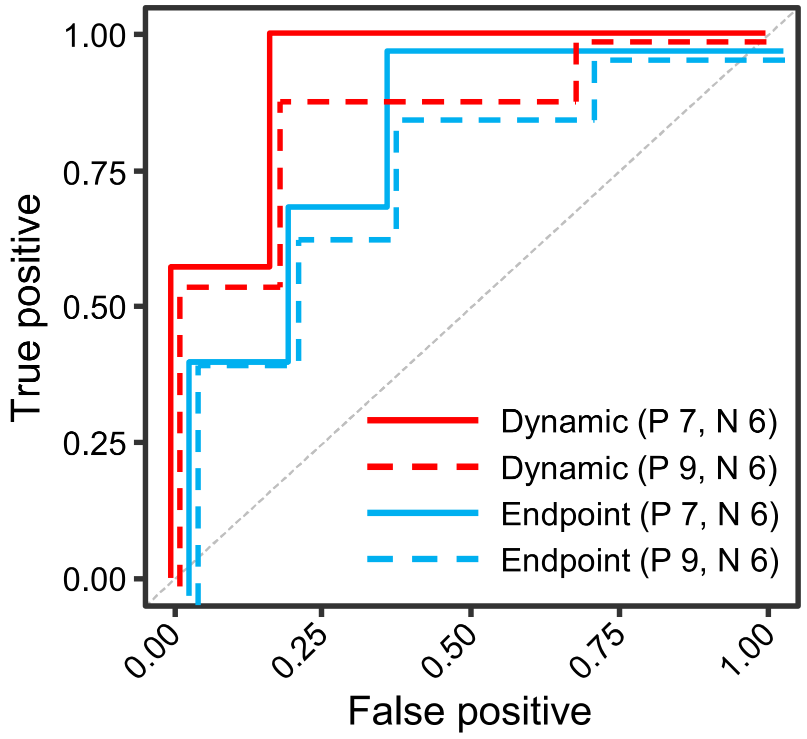

Assay	AUC	Threshold	Sensitivity	Specificity	Accuracy
Dynamic (P 7, N 6)	0.93	37.89	1.00	0.83	0.92
Dynamic (P 9, N 6)	0.87	37.89	0.89	0.83	0.87
Endpoint (P 7, N 6)	0.86	3.11	0.43	0.83	0.62
Endpoint (P 9, N 6)	0.82	1.95	0.78	0.67	0.73

Comparisons of ROC curves when 6AN and BrdU are categorized as either non-limb developmental toxicants (P 7, N 6) or limb malformation chemicals (P 9, N 6). The same calculations were performed for the single endpoint assay, in which the difference in the luminescence intensity at 24 h between the vehicle control group and chemical exposure group was measured instead of the ABC. The table at the bottom summarizes the performance of the dynamic and single endpoint assays. The values next to P and N indicate the number of limb malformation chemicals and non-developmental toxicants used for the analysis, respectively.

Supplementary Table 1. Frequency of appearance of chemical-related words in abstracts of 695 articles found with the keywords “limb and digit deformities” in the PubMed database, Related to STAR Methods.

Chemicals mentioned in association with limb / digit deformities	Chemical name frequency
Thalidomide	367
† RA	190
† Retinoic	110
Ethanol	62
Cyclophosphamide	52
† HU	50
Cd	46
Misoprostol	46
† VPA	44
† Methotrexate	43
† Retinoid	43
TCDD	43
† Retinoids	37
GSH	36
† Hydroxyurea	36
MNU	33
2-ME	31
† MTX	30
Acetazolamide	27
† MAA	27
† 2-MAA	26
† ASA	26
Cadmium	24
LSD	24
Ara-C	23
Caffeine	23
† Valproic	23
† All-*trans*-retinoic	22
Folic	21
Phenytoin	21
5-FU	19
BPA	18
Cocaine	18
Isotretinoin	18
Thalidomide-induced	18
Alkaloid	17
Dex	17
EGME	17
d-AZA	16
DEHP	16

† Mercury	14
Methylnitrosourea	14
4-Hydroperoxycyclophosphamide	13
Cholesterol	13
Leflunomide	13
† Aspirin	12
BSO	12
Etretin	12
ETU	12
Glutathione	12
Phthalate	12
† RA-induced	12
TTNPB	12
5-AZA	11
TBZ	11
13-*cis*-RA	10
6-MPr	10
Adriamycin	10
Ammodendrine	10
† ATRA	10
EMS	10
Olaquindox	10

Daggers (†) indicate the chemicals used in the ECVAM International Validation Study of embryotoxicity tests *in vitro*.

Supplementary Table 2. Chemicals suspected of inducing limb/digit deformities, Related to Table 2.

Chemicals	Species	Location of malformation	Reference
ATRA	mouse	Changes in the size and shape of the humerus, radius, and ulna.	Campbell et al. (2004)
	mouse	Limb disorders	Okuda et al. (1997)
HU	mouse	Limb reduction, digit anomalies	Schlisser and Hales (2013)
MTX	rabbit	Phocomelia, hemimelia, syndactyly, and ectrodactyly	Hyoun et al. (2012)
	human	Short limbs, talipes, hypodactyly, and syndactyly	Hyoun et al. (2012)
MAA	mouse	Digit malformations	Haryono et al. (2011)
	rat	Limb malformations	GHS Classification Result (Access in 2021)
	mouse	Digit and paw malformations	GHS Classification Result (Access in 2021)
MeHg	mouse	Missing limbs	Su and Okita (1976)
SA	rat	Pes varus	Tanaka et al. (1973)
	rat	Absence of forelimb buds	Joschko et al. (1993)
VPA	human	Limb malformation	Rodríguez-Pinilla et al. (2000)
	mouse	Limb abnormalities	Paradis and Hales (2013)
